# The appropriate management algorithm for diabetic foot: A single-center retrospective study over 12 years

**DOI:** 10.1097/MD.0000000000011454

**Published:** 2018-07-06

**Authors:** Jung Woo Chang, Woong Heo, Matthew Seung Suk Choi, Jang Hyun Lee

**Affiliations:** Department of Plastic and Reconstructive Surgery, Hanyang University Guri Hospital, Hanyang University College of Medicine, Guri, Korea.

**Keywords:** algorithm, diabetic foot, diabetic ulcer, management

## Abstract

**Background:**

Diabetic foot management is a challenge for reconstructive surgeons because it combines dramatically decreased circulation and chronic infection. The goal of managing this condition is to maximize viable tissue; however, unsatisfactory results, such as extremity amputation, are unavoidable in some cases. For appropriate management, thorough understanding of diabetic foot and the phased approach to its management is needed. The purpose of this study is to introduce an optimal algorithm for diabetic foot management by analyzing cases >12 years.

**Methods:**

A total of 274 patients with diabetic foot at Hanyang University Guri Hospital from 2005 to 2017 were reviewed. The management process was divided into 5 steps: patient evaluation, wound preparation, improving vascularity, surgery and dressing, and rehabilitation. Patient evaluation included a microbial culture, evaluation of vascularity, and an osteomyelitis assessment. During wound preparation, debridement and negative-pressure wound therapy were performed. Vascularity was improved by radiological intervention or surgical method. Surgery and dressing were performed depending on the indications. Rehabilitation was started after complete wound healing.

**Results:**

An infection was confirmed in 213 of 263 patients (81.0%). Of 74 cases in which a vascular study was performed, 83.8% showed arterial occlusion. When surgery was performed with complete eradication of the infection in 155 patients, the rate of revision surgery was 20.6%. The revision rate after surgery with a remnant infection of 66 patients was 40.9% (*P* = .0003). When surgery was performed after successful revascularization for improving blood flow of 47 patients, the rate of revision surgery was 21.3%. In contrast, the revision rate after surgery with unsuccessful or no revascularization of 174 patients was 28.2% (*P* = .359).

**Conclusion:**

Diabetic foot is a debilitating disease arising from multifactorial process. As its management is complex, a comprehensive but accessible treatment algorithm is needed for successful results. For this reason, the appropriate algorithm for diabetic foot management introduced in this study is significant.

## 1 Introduction

The prevalence of diabetes mellitus is increasing. Diabetes mellitus may be accompanied by dangerous complications, such as wound occurrence on the lower extremity, known as diabetic ulcer or diabetic foot. If a diabetic patient has vasculopathy and neuropathy, it is easy for a wound on the extremity to develop.^[1]^ Although such wounds occur on the foot through repeated irritation, the patient is not able to recognize it because of sensory deficits. Moreover, the unrecognized wound often becomes chronically unhealed, due to poor vascularity, which interferes with wound healing.^[2]^ The decreased circulation also forms an environment in which microorganisms can survive.^[3]^ As a result, diabetic foot is often observed in the form of a chronic wound with chronic infection.

Treating diabetic foot is a challenge for reconstructive surgeons. Poor vascularity results in unsatisfactory outcomes, and chronic infection induces recurrence of the wound. In some severe cases, reconstruction cannot even be attempted, and amputation is unavoidable.^[4]^ The goal of diabetic foot management is to minimize nonviable tissue and to maximize viable tissue within the wound. This enables the extremity to be salvaged by minimizing the amputation level. The final step is to return the patient to an ambulatory state.

Diabetic foot cannot be cured using the ordinary approach for wounds in nondiabetic patients. For the successful reconstruction of diabetic foot, a comprehensive approach including vascular evaluation and infection control should be applied.^[5–7]^ The management should be divided into detailed steps from evaluation of the patient to rehabilitation, and each step should be specialized.^[8]^

The aim of this study is to introduce an appropriate management algorithm for diabetic foot. The author reviewed and analyzed the diabetic foot cases treated at a single institution >12 years, and established the algorithm by categorizing the management steps. As previous studies have not suggested any systemic and simplified algorithm for diabetic foot management, this study is a significant step forward.

## 2 Materials and methods

This study was conducted in conformity with the World Medical Association Declaration of Helsinki, and the protocol was approved by the Institutional Review Board of Hanyang University Guri Hospital (IRB No.: 2017-11-011-002). Patients with diabetic foot who were admitted and managed between May 2005 and May 2017 at the Department of Plastic and Reconstructive Surgery of Hanyang University Guri Hospital were reviewed. All the patients eligible for study inclusion were hospitalized for diabetic foot with grade 2 to 5 in the Wagner grading criteria and patients were excluded those with Wagner grade 0, 1.

**Figure 1:**
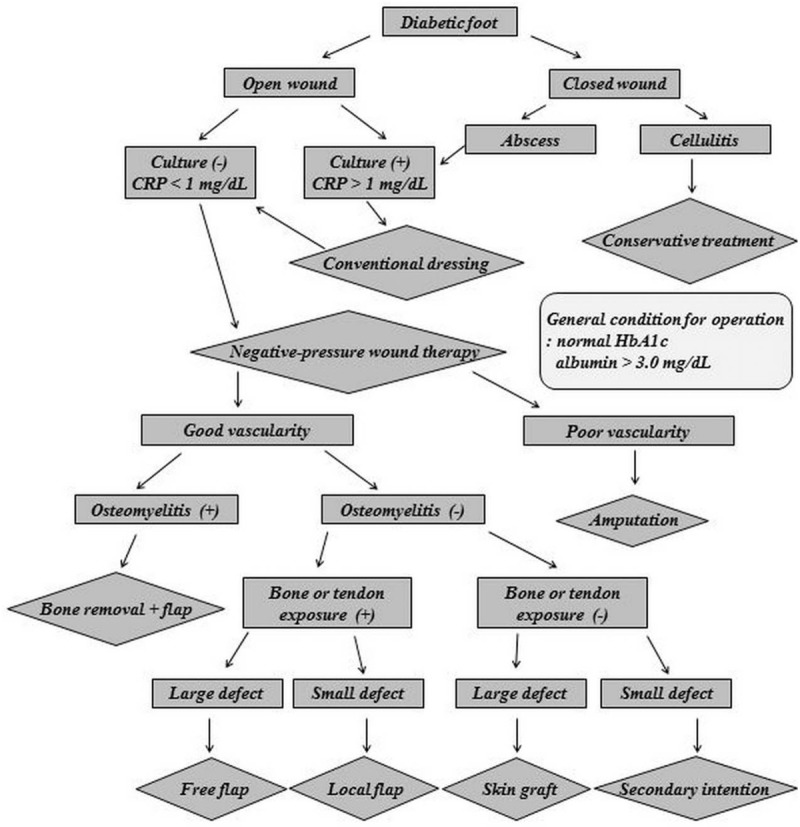
Algorithm for diabetic foot management. CRP = C-reactive protein, HbA1c = hemoglobin A1c.

To establish a standardized protocol, the management should be divided into 5 steps. The steps consisted of patient evaluation, wound preparation, improving vascularity, dressing and surgery, and rehabilitation. The optimal algorithm of diabetic foot management that the authors developed is presented in Figure 1. This algorithm contains each step of management and the decisions that are made at each step according to the patient's condition.

### 2.1 Patient evaluation

**Figure 2:**
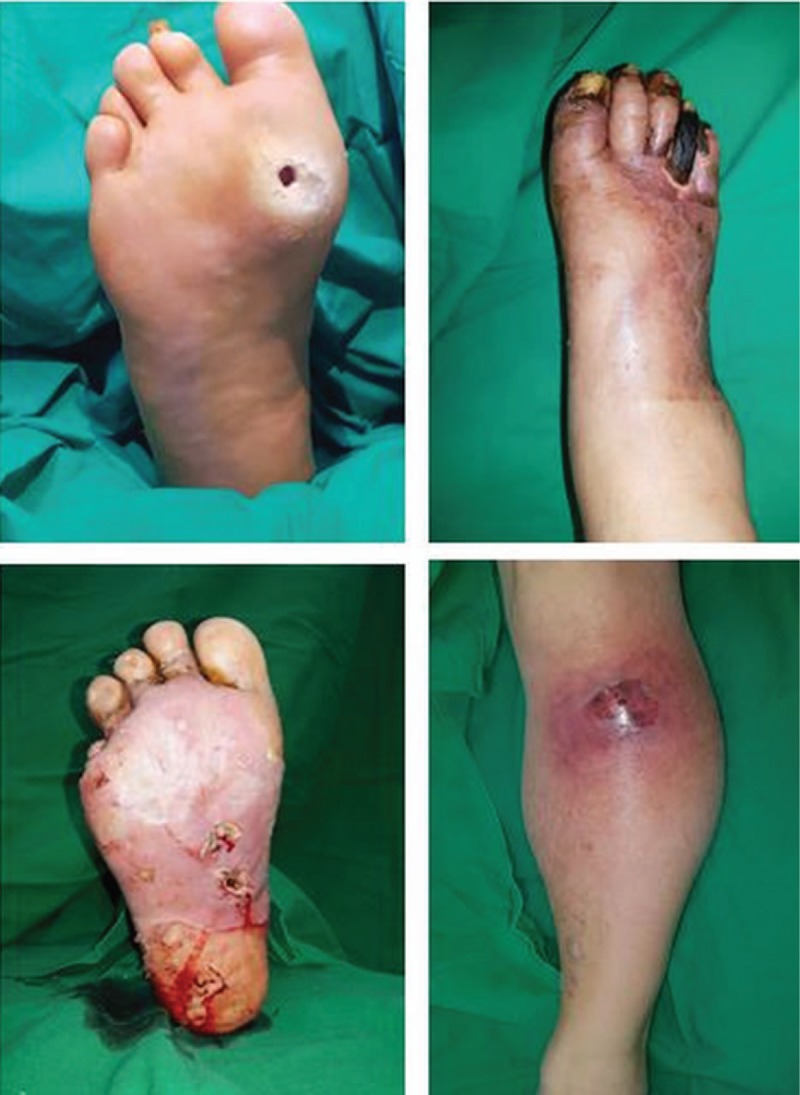
Various features of diabetic foot. (Above left) Diabetic foot with an open wound. (Above right) Diabetic foot with dry gangrene. (Below left) Diabetic foot with abscess formation within an open wound. (Below right) Diabetic foot with abscess formation within a closed wound.

**Figure 3:**
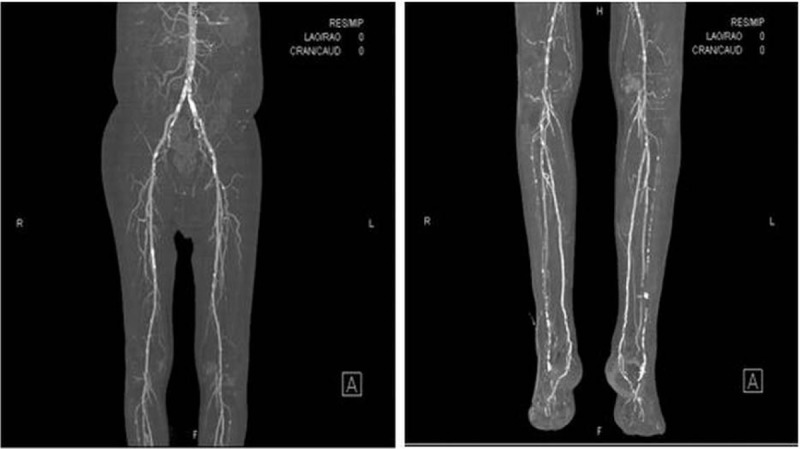
Computed tomography (CT) angiography of diabetic foot. (Left) CT angiography of the thigh. (Right) CT angiography of the lower leg.

When a patient presents with diabetic foot, the first step is to evaluate the wound. In most cases, an open wound is observed (Fig. 2), and a sample for microbial culture is obtained by a surface swab. The sample is sent to the hospital's microbiology laboratory. To evaluate whether osteomyelitis is present in the underlying bony structure, an X-ray study is performed, and additional studies such as a magnetic resonance imaging (MRI) or a bone scan can be performed if needed. If there is no open wound, and only signs of infection such as redness and swelling are observed (Fig. 2), computed tomography (CT) should be performed because CT imaging allows abscess formation to be distinguished from simple cellulitis. For evaluating vascular insufficiency, CT angiography is performed (Fig. 3). The entire lower extremity is evaluated to localize the level of vessel occlusion in the external iliac artery, femoral artery, anterior tibial artery, posterior tibial artery, or peroneal artery.

### 2.2 Wound preparation

**Figure 4:**
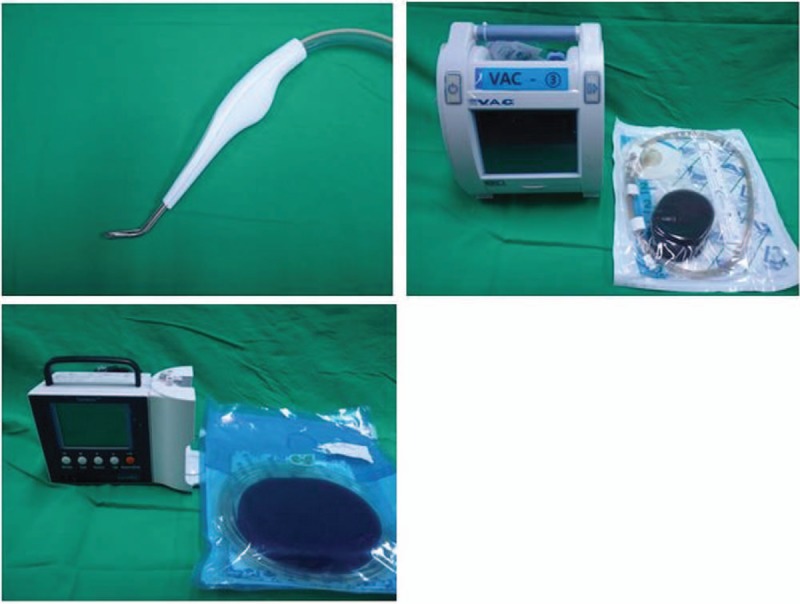
Devices for wound preparation. (Above left) Hydrosurgery device (Versajet, Smith & Nephew, UK). (Above right) Negative-pressure wound therapy (NPWT) device (V.A.C., KCI, Germany). (Below) NPWT device (CuraVac, CGBio, Korea).

The goal of this step is to make the wound suitable for surgery. A wound with clean granulation in which the infection is eradicated is ready for surgery. As chronic infection is common, the biofilm formed by microorganisms on the open wound surface should be removed by curettage. When necrotic or nonviable tissue is observed, surgical debridement is performed. In cases of radical debridement, a hydrosurgery device (Versajet, Smith & Nephew, UK) can be used (Fig. 4). All bones with osteomyelitis are removed if possible. If the wound is not infected, as confirmed by the absence of growth in the culture, negative-pressure wound therapy (NPWT) is applied to induce granulation. NPWT devices vary [(V.A.C., KCI, Germany) (CuraVac, CGBio, Korea)], but are equivalent in terms of treatment indications (Fig. 4). If the wound is infected, as indicated by a positive culture result, NPWT is not applied until the infection is eradicated. For complete eradication, betadine soaking or betadine wet dressing is applied to the infected wound. During the dressing period, intravenous antibiotics suitable to the microorganisms presented in the wound are administered. When infection was controlled clinically, NPWT is applied. Complete eradication is confirmed by a routine microbiological study with no growth of bacteria. With a closed wound, if abscess formation is confirmed by a CT scan, surgical drainage is performed. During complete drainage, a sample for microbial culture is obtained from the abscess. After drainage, the wound is left opened for serial irrigation and dressing. The open wound is closed or reconstructed with other options in a later step.

### 2.3 Improving vascularity

**Figure 5:**
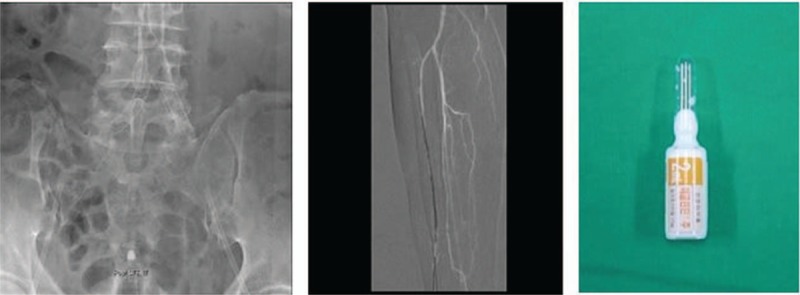
Tools for improving vascularity. (Left) Revascularization by stent insertion. (Middle) Revascularization by balloon angioplasty. (Right) Prostaglandin E1 (Eglandin, Mitsubishi, Japan) for vasodilation.

The goal of this step is to improve the decreased blood flow in the extremity. This step is conducted simultaneously with wound preparation. After evaluation of the vessel occlusion level by CT angiography, the occluded site needs to be revascularized. If the occlusion level is above the knee, a stent is inserted at the occluded site through a radiological intervention. In severe cases in which a stent is not applicable, endarterectomy surgery is performed to improve blood flow. If the occluded site is below the knee, balloon angioplasty is performed through a radiological intervention. After the radiological intervention or vascular surgery, aspirin is administered to prevent thrombus formation, and prostaglandin E1 (Eglandin, Mitsubishi, Japan) is administered intravenously for vasodilation effect. If occlusion is not observed or not severe, prostaglandin E1 is administered intravenously as a vasodilator, with no other intervention (Fig. 5).

### 2.4 Surgery and dressing

After controlling the wound condition, surgery is performed. In addition to the wound condition, the patient's general conditions, including factors such as glycemic control and nutritional status, are considered when deciding the optimal time for surgery. Hemoglobin A1c should be controlled in the normal range, between 4.4% and 6.4%, and albumin level should be maintained at a level >3.0 mg/dL before surgery.

The choice of the surgery procedure depends on the presence of osteomyelitis and the circulatory condition of the extremity. As osteomyelitis is the main source of wound recurrence, bone affected by osteomyelitis should be removed. Poor blood supply continuing after the failure of a vascular intervention is also incurable.

If the circulation in the extremity is severely impaired, the extremity is amputated at the most proximal level where fair circulation is observed. Fair circulation is confirmed by the observation of adequate bleeding during the operation. According to the level of amputation, various operations are performed, including interphalangeal (IP) disarticulation, metatarsophalangeal (MTP) disarticulation, ray amputation, Lisfranc amputation, Chopart amputation Syme amputation, below-knee (BK) amputation, and above-knee (AK) amputation.

**Figure 6:**
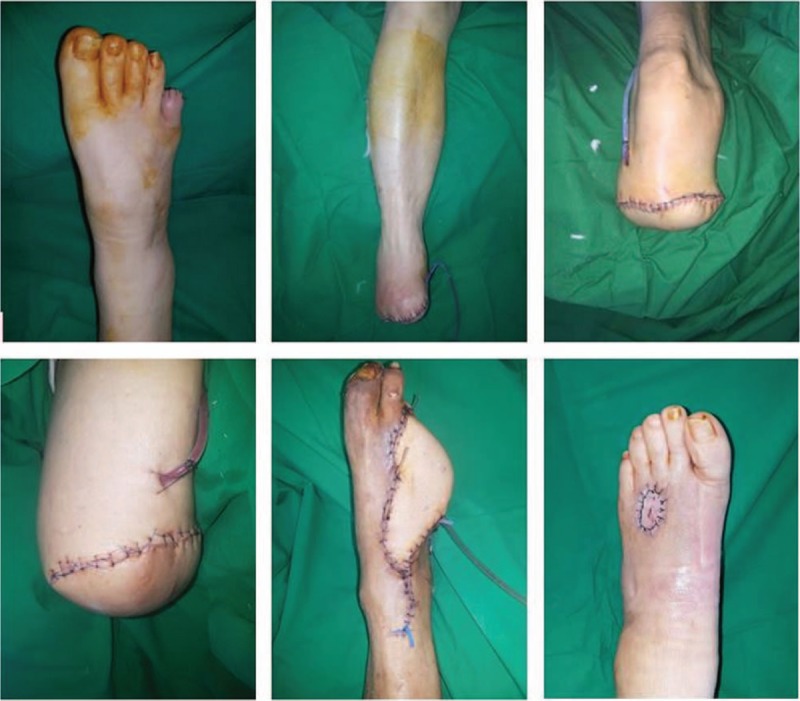
Surgical options for diabetic foot treatment. (Above left) Interphalangeal disarticulation. (Above middle) Lisfranc amputation. (Above right) Below-knee amputation. (Below left) Above-knee amputation. (Below middle) Free flap coverage. (Below right) Split-thickness skin graft with acellular dermal matrix.

If osteomyelitis is present despite a good circulatory condition, bone removal is performed and the wound is covered with a flap. A free flap is applied to a large wound, whereas a local flap is applied to a small wound. If there is no osteomyelitis and the circulatory condition is good enough for salvage, various forms of reconstructive surgery can be performed. The choice of the surgical option depends on whether bone or tendon exposure is seen. If bone or tendon is exposed within the wound, flap surgery is performed. Whether a free flap or local flap is used depends on the wound size. If there is no bone or tendon exposure, the wound is treated with a skin graft or by secondary intention (Fig. 6).

Secondary intention, which refers to healing with a wound dressing, is applied to small wounds without bone or tendon exposure. Supplementary materials, such as epidermal growth factor (EGF), collagen, and polydeoxyribonucleotide (PDRN), are used for dressing refractory wounds. The end point of wound healing is considered as totally epithelized wound without any discharge.

### 2.5 Rehabilitation

**Figure 7:**
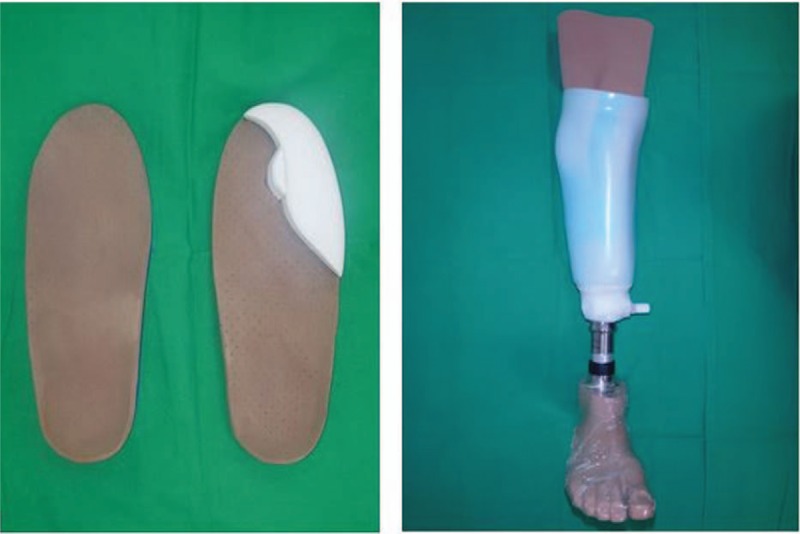
Supplements for rehabilitation. (Left) Customized insole of shoes for an amputated foot. (Right) Prosthesis for a patient who underwent below-knee amputation.

The rehabilitation process begins when the wound is completely healed. The aim of rehabilitation is to enable the patient to walk again, and it is conducted by physicians specializing in rehabilitation. If amputation is performed, specialized equipment such as customized shoes or prostheses are used to help patients recover ambulation (Fig. 7). The end point of rehabilitation is considered when patients can walk for an hour or more.

### 2.6 Statistical analysis

Statistical analysis was performed using SPSS 18.0 (SPSS Inc., Chicago, IL), and Fisher exact test was used to determine statistically significant differences in the revision surgery rate. A *P* value of <.05 was considered statistically significant.

## 3 Results

Our population of 274 patients consisted of 168 males and 106 females. Their mean age was 64.4 years (32–87 years), and the average follow-up period was 18.8 months (9–28 months). Among the 274 patients, 250 had a soft tissue defect on their extremities, whereas 24 had closed wound. All 24 patients with a closed wound underwent a CT scan to determine whether an abscess had formed, and in 13 patients, abscess formation was identified. They all underwent immediate surgical drainage of the abscess with the wound left open. The wound states of 250 patients with an open wound ranged from the third to fifth grades of the Wagner Grading Criteria (WGC). In 24 patients with a closed wound, 13 patients with an abscess formation were third grade of WGC and 11 patients with no abscess were second grade of WGC (Table 1). For the 250 patients with an open wound and the 13 patients with an abscess within a closed wound, a culture was performed to identify microorganism in the wound. A causative organism was identified in 213 (81.0%) of these 263 cases. From the 213 infected wounds, 300 microbial isolates were confirmed (Table 2). During the wound preparation period, all 213 infected open or closed wounds of 263 patients (250 patients with an open wound and 13 patients with an abscess within a closed wound) were managed through the intravenous administration of suitable antibiotics, debridement, abscess drainage, and wet dressing. NPWT was applied in 52 cases after the control of the infection. The average preparation period of the infected wounds for the surgical procedures was 15.1 days. The infection was eradicated completely before surgery in 155 patients and eradicated incompletely in 66 patients. Of the 50 noninfected wounds, 31 underwent NPWT. The average preparation period of the noninfected wounds for the surgical procedures was 7.2 days. The 11 patients with no abscess within a closed wound were treated with intravenous antibiotics and dressing.

**Table 1:**
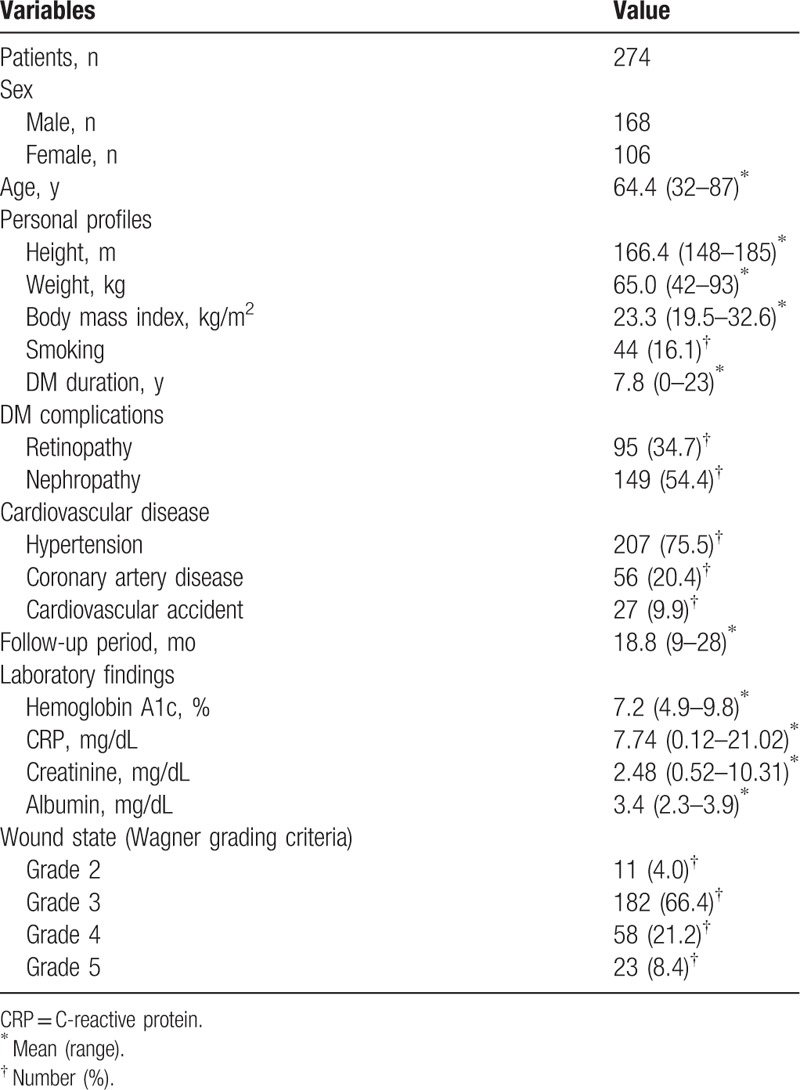
Patient information.

**Table 2:**
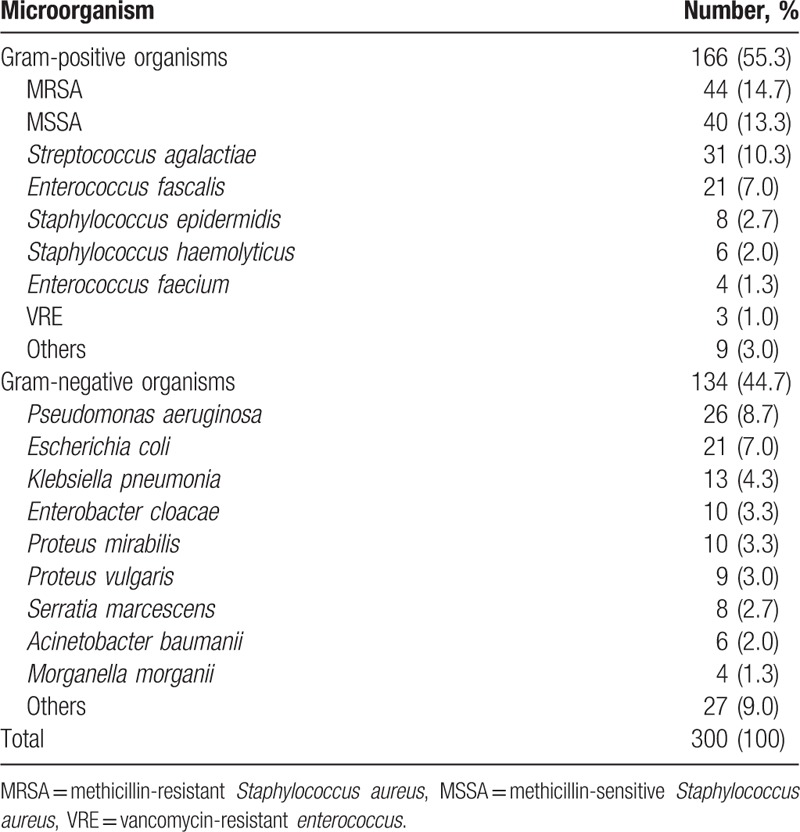
Microorganisms found in cultures from cases of diabetic foot.

An X-ray study was performed to evaluate the bone status of 256 patients, and osteomyelitis was observed in 155 patients. In 19 cases, an MRI study was performed for further evaluation, and osteomyelitis was observed in 17 of those cases. A bone scan study was performed in 19 cases, 12 of which showed osteomyelitis.

CT angiography was done in 74 patients to identify vessel occlusion. Mild arterial stenosis not requiring an intervention was seen in 12 of those patients. The remaining 62 patients had arterial occlusion, for which radiological or surgical intervention was indicated. The level of the occlusion was above the knee in 12 patients, and below the knee in 50 patients. Six of these 62 patients declined the intervention for financial reason. Fifty-four patients had a consultation with a radiologist at our institution regarding a radiologic intervention, and 33 patients underwent arterial ballooning in the occluded arteries at BK level, among the anterior tibial artery, posterior tibial artery, and peroneal artery. Twelve patients underwent stent insertion in the occluded arteries at AK level, including femoral artery and external iliac artery. They all showed improved arterial flow after the intervention. An intervention was attempted in the remaining 9 patients, but the intervention failed because of their poor vascular condition. Two patients with severe proximal occlusion had a consultation with a general surgeon at out institution regarding a surgical intervention. They underwent endarterectomy surgery at the occluded site in femoral artery, and the arterial flow improved (Table 3).

**Table 3:**
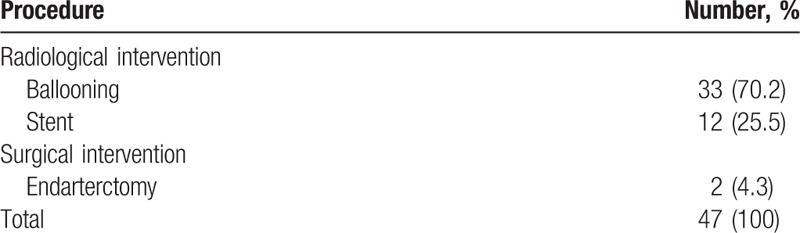
Revascularization procedures performed to improve vascularity.

A total 280 cases of surgery were performed in 221 of 274 patients and 59 revision surgery. Depending on the wound condition, a choice was made between the surgical options such as amputation, flap coverage, or skin graft. (Table 4). Healing without surgery, which means healing by secondary intention alone, took place in 53 patients.

**Table 4:**
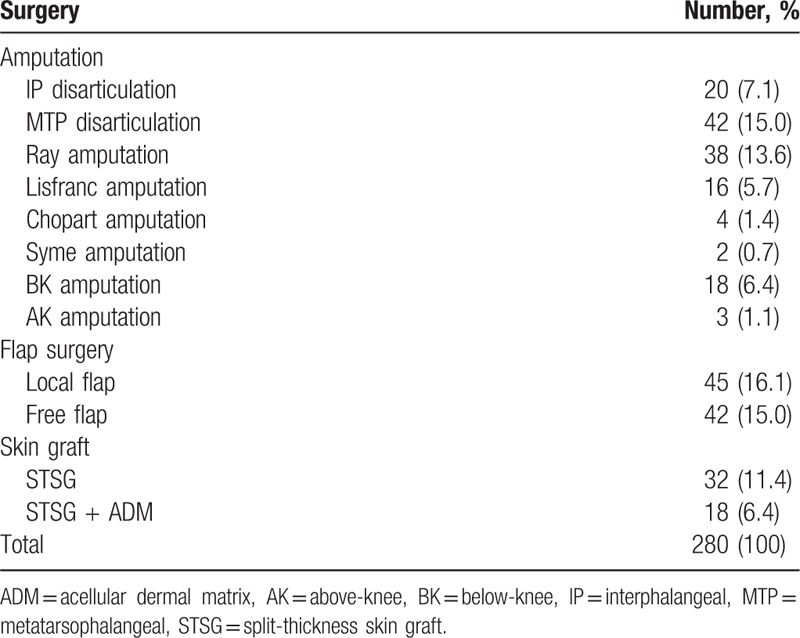
Surgical procedures performed in patients with diabetic foot.

Fifty-nine revision procedures were additionally performed in 221 patients. Revision was deemed necessary when necrosis or infection was observed at the operated site, and the overall revision rate was 26.7%. When surgery was performed after complete eradication of the infection, the revision rate was 20.6%, whereas surgery performed without complete eradication of the infection had a revision rate of 40.9%. This result was statistically significant (*P* = .003). When surgery was performed after MRI or bone scan study, the revision rate was 15.8%, whereas surgery performed in patients who did not undergo MRI or bone scan study had a revision rate of 30.0%. The revision rate of surgery after a vascular intervention was 21.3%, whereas that of surgery without a vascular intervention was 28.2%. These results were not statistically significant (*P* = .108 and .0359, respectively) (Table 5).

**Table 5:**
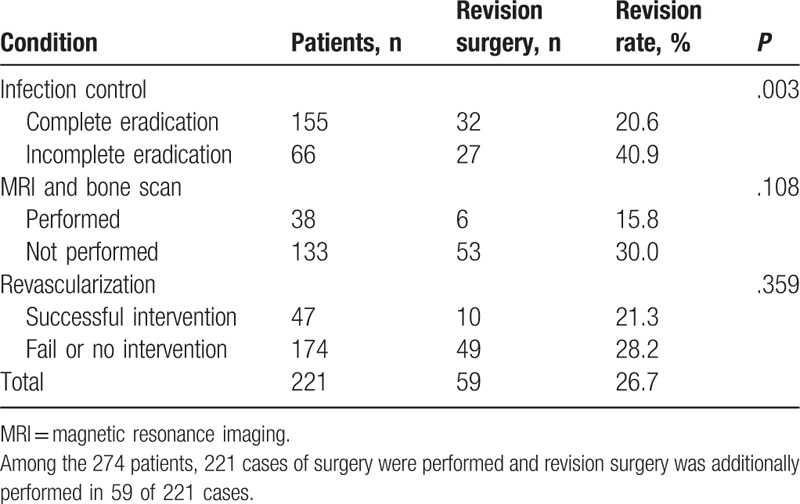
Revision surgery rate.

A total of 177 patients underwent rehabilitation for ambulation. Rehabilitation was provided to patients who were able to walk before surgery. The average time required from surgery to rehabilitation was 24.4 days. The ambulation rate (i.e., the rate of patients who could walk preoperatively and recovered ambulation after surgery) was 72.3%.

## 4 Discussion

Diabetic ulcers are delicate wounds. Appropriate management requires the surgeon to carefully consider vascular insufficiency and chronic infection.^[9,10]^ Without adequate consideration of the character of diabetic foot, management often fails.

Infections in diabetic foot are common and should be treated aggressively.^[11]^ In this study, 81.0% of the patients had an infection, as confirmed by the initial microbial culture, and 44.1% of them were infected with multiple pathogens. The ratio of gram-positive organism to gram-negative organism was 55 to 45, and the most common organism in each group was Methicillin-resistant *Staphylococcus aureus* (14.7%) and *Pseudomonas aeruginosa* (8.7%), respectively. These results demonstrate a similar tendency to other studies, but different proportions. In a large review study of a Western population in 2011, the proportion of gram-positive organisms was higher (60%–77%) than our results.^[12]^ In that study, the most common gram-positive organism was *S. aureus* (6.5%–48.8%), mostly methicillin-sensitive *S. aureus*, and the most common gram-negative organism was *Enterobacteriaceae* (7.0%–33.7%). The proportion of *P. aeruginosa* was 2.5% to 14.6%. Although the present study found that gram-positive organisms were more common than gram-negative organisms in our hospital, the proportion was lower than was reported in a Western population. In addition, the most common pathogens found in our study are more difficult to eradicate than those reported in that Western population. Such differences have also been observed in various regions, such as the Middle East and Africa. The relative frequencies of causative organisms of diabetic foot may vary regionally.^[13,14]^

Through this analysis, the most common causative organisms in our hospital were identified, which is useful information for developing an optimal regimen for empirical antibiotic use. As infection control is important in diabetic foot management, it is necessary to use empirical antibiotics during the initial management, before the culture confirmation.^[15]^ However, the regimen should be established according to local conditions due to variability in the prevalence of specific microorganisms.

The importance of infection control is shown by the revision surgery rate. When surgery was performed with a remnant infection, the revision rate was 40.9%. In most cases, the revision was performed because of wound rupture induced by the remnant infection. When surgery was performed without an MRI or bone scan study, the revision rate was 30.0%. This may have resulted from undiagnosed osteomyelitis that was missed in X-ray studies.

Decreased vascularity is another important factor to consider. Arterial stenosis in diabetic foot is common, and our study showed 83.8% of the patients who underwent CT angiography. Low blood flow induces delayed wound healing and leads to poor postoperative results.^[2]^ For this reason, patients’ vascular status should be assessed as part of their evaluation. If stenosis is observed, endovascular treatment for improving the blood flow is needed.^[16]^ The effectiveness of revascularization on ischemic ulcer healing remains controversial. In 2011, Taylor et al reported that there was no statistically significant difference in outcomes between patients with ischemic ulcers that were revascularized as compared with ischemic ulcers that were not revascularized.^[17]^ However, in our study, revascularized patients showed better wound healing with a revision rate of 21.3% than patients without revascularization who had a revision rate of 28.2%. Improved blood flow may result in better wound healing, which prevents reamputation or flap revision.

During the last decade, a tremendous increase has been seen in endovascular devices and techniques to treat vascular occlusive disease.^[18]^ Percutaneous transluminal angioplasty (PTA) is a widely used method, with a range of subtypes including balloon angioplasty, drug-eluting balloon angioplasty, and stents.^[19]^ Generally, for dilation below the knee level, only balloon angioplasty is applied regardless of vascular condition. For dilation above the knee level, balloon angioplasty is applied in case of mild occlusion, whereas a stent is used for severe occlusion. Drug-eluting balloon angioplasty is a good option, but is not used in our hospital because of its high cost and difficulties regarding insurance coverage.

Surgical revascularization procedures such as endarterectomy and bypass surgery have been introduced.^[20]^ Previous studies have reported that these techniques had good salvage rates in case of ischemic foot. In our study, endarterectomy was performed in severely occluded cases that could not be improved by PTA. Endarterectomy is more invasive than PTA and should be performed under general anesthesia. Nevertheless, surgical methods are still effective for refractory occlusions as a last option.

It is important to choose an appropriate surgical method for wound reconstruction. In suitable preoperative conditions, such as clean granulations without infection, surgical coverage of the defect is appropriate. In the past, amputation was widely performed in diabetic foot. However, as microsurgical skills have developed, free tissue transfer can now allow extensive amputation to be avoided.^[21]^ Removing bones with osteomyelitis is unavoidable,^[22]^ but free tissue transfer can save soft tissue as much as possible. As a result, the length of the extremity can be preserved to the greatest extent possible.

Whether bones with chronic osteomyelitis should be resected remains a topic of debate. Recently, some have disputed whether routine surgical resection is appropriate, arguing that resecting bones may run the risk of architectural reorganization of the foot, resulting in altered biomechanics and additional ulceration.^[23,24]^ Based on this theory, nonsurgical treatments with a prolonged course of antibiotics have been tried.^[25]^ However, these studies often failed to provide a precise definition of osteomyelitis. Furthermore, in our experience, complete eradication of osteomyelitis with nonsurgical treatment was impossible, and remaining infected bone was the primary source of wound recurrence, especially in the phalangeal and metatarsal bones.

Sufficient vascularity must be present before flap surgery. As vascularity affects flap survival, flap surgery should not be performed in patients with poor circulation. If sufficient vascularity is not observed, amputation at the lowest level where there is enough circulation is unavoidable. Recently, however, supermicrosurgery has emerged as another option in cases with severe vascular occlusion.^[26]^ Flap surgery with supermicrosurgery is based on anastomosis at the perforator level.^[27]^ The use of this procedure is based on the theory that small branch vessels are found to be uninvolved in the premature atherosclerosis that individuals with diabetes suffer from.^[28]^ As this option is still risky, it should be chosen as a last option can be applied.

The main advantage of flap surgery is that it provides durability with sufficient amount of tissue. For this reason, flap coverage is effective in areas with bone exposure and in weight-bearing areas. Areas with tendon exposure where a skin graft cannot be used are also good candidates for flap coverage. If the defect is small, a local flap from an adjacent area can be applied. A free flap is suitable for covering a large defect. Otherwise, a skin graft can be considered in areas without bone exposure or with nonweight bearing. Although grafted skin is less durable than a flap, it is good option to cover these areas. Recently, acellular dermal matrix has been widely used to add elasticity under split-thickness skin graft.^[29,30]^ As a form of artificial dermis, it supports the superficial skin.

Healing by secondary intention can be attempted only for small defects without bone exposure. This modality induces granulation and epithelialization by the dressing treatment. As diabetic ulcers do not respond well to ordinary dressings, supplementary materials should be added. Recently, EGF has been widely used to promote rapid wound healing.^[31]^ The authors prefer using the spray type. Spraying EGF solution on the raw surface of a diabetic ulcer induces granulation tissue formation and epithelialization. Collagen materials are also effective for rapid granulation.^[32,33]^ Various types, such as sheet and grinded forms, have recently entered use. They can be applied on the wound alone or with EGF. PDRN injection is another form of supplement.^[34,35]^ PDRN is a mixture of nucleotides, stimulating vascular endothelial growth factor production under low tissue-perfusion condition, as encountered in diabetes mellitus.^[36,37]^ It can be injected directly into the wound, but the authors prefer intramuscular injection in the patient's gluteal region. These supplementary materials are essential for managing refractory wounds such as diabetic ulcers in this manner.

In addition to wound care, glycemic control and nutrition are important factors for diabetic foot management. High glucose concentrations in the blood interfere with wound healing, and are correlated with various complications, such as cardiovascular disease, retinopathy, and nephropathy.^[38]^ For this reason, strict glycemic control is needed, and the measurement of plasma hemoglobin A1c, a standard metric of glycemic control, is essential.^[39]^ Hemoglobin A1c levels should be routinely checked every 4 months. Nutrition should be adequate to provide sufficient protein for the growth of granulation tissue.^[40]^ There is no accepted standard method for nutritional assessment, but serum albumin and prealbumin levels are widely used. Albumin levels reflect long-term protein consumption, whereas prealbumin levels reflect recent protein consumption. In this study, albumin levels were measured for nutritional assessment, and a level >3.0 mg/dL was maintained during wound treatment. However, for the accurate assessment of a patient's acute status, the prealbumin level should also be measured. Prealbumin is more reliable than albumin for assessing a patient's current nutritional status because of its short half-life of 2 days.^[41]^

The wound must be classified as open or closed. In closed wounds, abscess formation should be distinguished from simple cellulitis. Cellulitis is treated conservatively, whereas an abscess should be drained by surgical drainage and the infection should be eradicated before reconstructive surgery. In open wounds, the infection must be eradicated before reconstructive surgery, and the noninfected wound should be prepared with NPWT. The surgical option is chosen depending on the patient's circulatory status and whether osteomyelitis is present. With poor circulation, amputation is unavoidable, but if there is enough circulation for salvage, various reconstructive procedures can be attempted. If osteomyelitis is observed, flap surgery with removal of the affected bone should be performed. If no osteomyelitis is present, the options depend on whether bone or tendon is exposed through the wound. If bone or tendon is exposed, flap surgery should be considered. If there is no exposure, skin graft or dressing treatment can be considered. Rehabilitation should be started after complete wound healing.

The limitation of this study is that the efficacy and outcome of our algorithm of diabetic foot management could not be demonstrated because the algorithm could not be compared with an appropriate control group. Therefore, the authors compared our management strategies with the previous literatures. In recurrence rate, Armstrong et al reported that the recurrence rate is roughly 40% and Lavery ea al reported 8% to 59% within 1 year after surgery.^[41,42]^ Izumi et al reported that the rate of reamputation is 27%.^[43]^ In our study, the patients with complete eradication were 20.6%, the patients with MRI and bone scan were 15.8%, and the patients with revascularization were 21.3% in revision surgery rate. Consequentially, this results showed that the outcome of author's algorithm is better than the previous research.

Diabetic foot is a serious complication that lowers patients’ quality of life. The need for a prolonged hospital stay, the high treatment cost, and the high rate of lower-extremity amputation indicate the tremendous burden on diabetic patients. If lower-extremity amputation is performed, the patient may suffer not only from economic costs, but also from psychological stress. An inappropriate approach to diabetic foot management may exacerbate these difficulties. Therefore, to ensure an optimal management strategy, the reconstructive surgeon must understand the pathophysiology of diabetic foot. The most appropriate management strategy involves improving the vascularity and eradicating the infection. Moreover, the optimal selection of a surgical procedure and its skillful execution can enable possible foot salvage. The algorithm introduced in this study was established based on the author's accumulated experience. Although diabetic foot management is a complex process, the application of this comprehensive but simple algorithm may contribute to more successful results.

## Author contributions

**Conceptualization:** Jung Woo Chang, Matthew Seung Suk Choi, Jang Hyun Lee.

**Data curation:** Jung Woo Chang, Woong Heo, Jang Hyun Lee.

**Formal analysis:** Jung Woo Chang, Woong Heo.

**Investigation:** Jung Woo Chang, Woong Heo.

**Methodology:** Jung Woo Chang, Jang Hyun Lee.

**Supervision:** Jang Hyun Lee.

**Writing - original draft:** Jung Woo Chang.

**Writing - review & editing:** Jung Woo Chang, Woong Heo, Jang Hyun Lee.
